# Supplementary material for: Visualizing delocalized correlated electronic states in twisted double bilayer graphene
Source: Nat Commun. 2021 May 4;12:2516. doi: 10.1038/s41467-021-22711-1 (PMC8096954; doi:10.1038/s41467-021-22711-1)
Supplement: Supplementary file 1 — Supplementary Information [file 41467_2021_22711_MOESM1_ESM.pdf]

Supplementary information:

Visualizing delocalized correlated electronic states in twisted double bilayer graphene

Canxun Zhang,[1, 2, 3, 7] Tiancong Zhu,[1, 2, 7] Salman Kahn,[1, 2, 7] Shaowei Li,[1, 2, 3] Birui Yang,[1] Charlotte Herbig,[1] Xuehao Wu,[1] Hongyuan Li,[1, 2] Kenji Watanabe,[4] Takashi Taniguchi,[5] Stefano Cabrini,[6] Alex Zettl,[1, 2, 3] Michael P. Zaletel,[1, 2]* Feng Wang,[1, 2, 3]* and Michael F. Crommie[1, 2, 3]*

[1]Department of Physics, University of California, Berkeley, CA 94720, USA.

[2]Materials Sciences Division, Lawrence Berkeley Laboratory, Berkeley, CA 94720, USA.

[3]Kavli Energy NanoScience Institute, University of California, Berkeley, CA 94720, USA.

[4]Research Center for Functional Materials, National Institute for Materials Science, 1-1 Namiki, Tsukuba 305-0044, Japan.

[5]International Center for Materials Nanoarchitectonics, National Institute for Materials Science, 1-1 Namiki, Tsukuba 305-0044, Japan.

[6]Molecular Foundry, Lawrence Berkeley Laboratory, Berkeley, CA 94720, USA.

[7]These authors contributed equally.

*Correspondence to: mikezaletel@berkeley.edu (M. P. Z.); fengwang76@berkeley.edu (F. W.); crommie@berkeley.edu (M. F. C.)

Table of contents

Section	Title	Page
Note 1	Determination of three stacking orders	3
Note 2	Additional data on different devices and/or spots	6
Note 3	Comparison of STM/STS and transport measurements	8
Note 4	Extracting the splitting magnitude from d*I*/d*V* spectra	10

Note 5	Spatial distribution of layer-summed CFB and RCB wavefunctions	13
Note 6	Layer polarization of flat band wavefunctions in an *E*- field	16
Note 7	Screened Coulomb interaction in Hartree-Fock calculations	18
Note 8	Competition between different symmetry-breaking correlated states	18
Note 9	Tip-bias-induced gating	24
Figure 1	d*I*/d*V* spectroscopy showing phonon-mediated inelastic tunneling	4
Figure 2	Spatial distribution of valence flat band and remote valence band wavefunctions	5
Figure 3	Gate-dependent d*I*/d*V* spectroscopy on different devices and/or spots	7
Figure 4	Parameter space explored in STM/STS and transport measurements	9
Figure 5	Double Lorentzian fitting of the d*I*/d*V* signal	11
Figure 6	d*I*/d*V* spectroscopy for three stacking regions for $1 < \nu < 3$	12
Figure 7	Spatial distribution of layer-summed CFB and RCB wavefunctions	14
Figure 8	Gate-dependent LDOS calculated using a continuum model	15
Figure 9	Layer polarization of flat band wavefunctions in an *E*-field	17
Figure 10	Hartree-Fock phase diagram for $\nu = 2$	21
Figure 11	Hartree-Fock calculations for d_S = 50 nm and ε_{eff} = 14	22
Figure 12	Hartree-Fock calculations for d_S = 50 nm and ε_{eff} = 20	23
Figure 13	Theoretical LDOS including tip-bias-induced gating	25

Supplementary Note 1: Determination of three stacking orders

Our STM image of tDBLG in Fig. 1c shows three distinct regions within each moiré unit cell, which can be called “bright”, “intermediate” and “dark” based on their apparent heights for 100 mV < $|V_{\mathrm{Bias}}|$ < 500 mV. We determined their stacking orders by analyzing structural as well as electronic contributions to the apparent height. In tDBLG, ABBC stacking is energetically unfavorable (due to the strong repulsion between inner layers of carbon atoms) and has been predicted to exhibit an out-of-plane structural displacement of ~0.1 Å.[1] This allows us to identify the “bright” region in Fig. 1c as ABBC stacked. ABAB and ABCA stackings, on the other hand, have comparable binding energies and similar structural heights. Atomic structure alone is thus inadequate to explain the difference between the observed “intermediate” and “dark” regions. However, if we calculate the LDOS at the ABAB and ABCA sites using the continuum model and integrate them from –200 to 0 meV (simulating a negative V_{Bias}) or from 0 to 200 meV (simulating a positive V_{Bias}), ABCA always displays a higher intensity than ABAB. Since a larger integrated LDOS corresponds to a higher apparent height in constant-current STM measurements, we identify the “dark” region as ABAB stacked and the “intermediate” region as ABCA stacked.

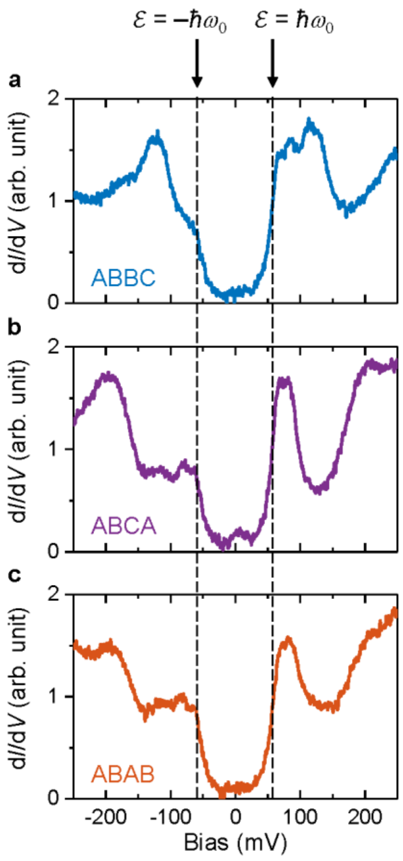

Supplementary Figure 1: d*I*/d*V* spectroscopy showing phonon-mediated inelastic tunneling. a-c, d*I*/d*V* spectra for the three stacking regions at $V_G = 0$ V (modulation voltage $V_{RMS} = 2$ mV; initial $V_{Bias} = –250$ mV, $I_0 = 0.1$ nA). The dashed lines mark the energy of the phonon mode and the onset of the inelastic channel.

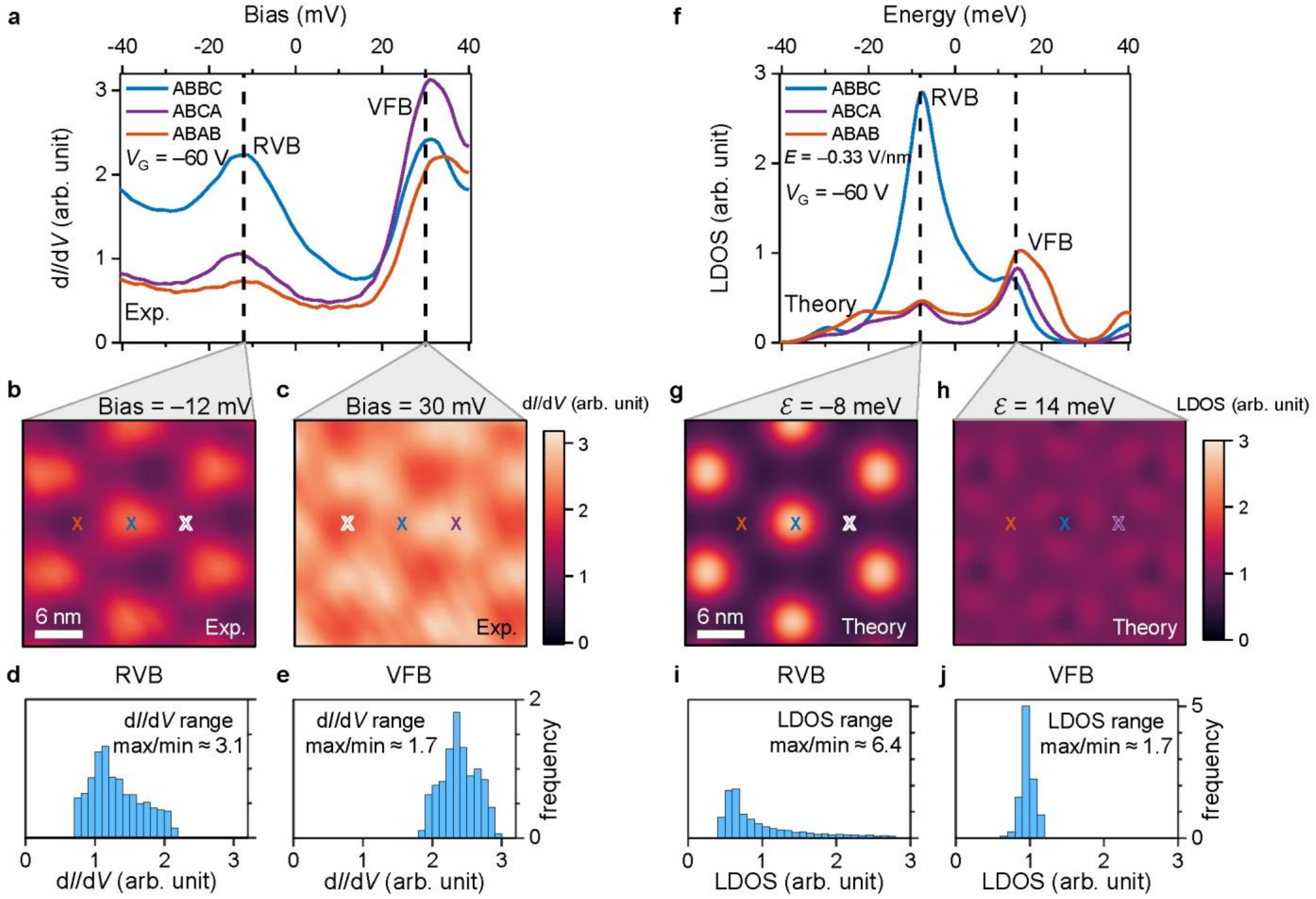

Supplementary Figure 2: Spatial distribution of valence flat band and remote valence band wavefunctions. a, dI/dV spectra for three stacking regions measured at V_G = –60 V. **b, c**, dI/dV maps of the same tDBLG region obtained at (**b**) V_{Bias} = –12 mV and (**c**) V_{Bias} = 30 mV (modulation voltage V_{RMS} = 1 mV; initial V_{Bias} = –500 mV, I_0 = 2.5 nA). **d**, Histogram of (**b**). **e**, Histogram of (**c**). **f**, Theoretical LDOS of three different stacking regions for V_G = –60 V and E = –0.33 V/nm calculated using a single-particle continuum model. **g**, **h**, LDOS maps at (**g**) –8 meV and (**h**) 14 meV. **i**, Histogram of (**g**). **j**, Histogram of (**h**). All histograms use bin size of 0.1 arb. unit. Areas under histograms are normalized to 1. VFB = valence flat band; RVB = remote valence band.

Supplementary Note 2: Additional data on different devices and/or spots

The LDOS suppression around $\nu = 2$ was observed on different devices and/or spots, as shown in the gate-dependent dI/dV spectra of three stacking sites in Supplementary Fig. 3. Here, the first set of data was taken on the same device as the data shown in the main text, but at a different spot on the surface (micrometers away) with a different local twist angle (a-c), while the second set was taken on a different device. The STM tips were freshly prepared and calibrated on Cu(111) before each set of measurements (Methods).

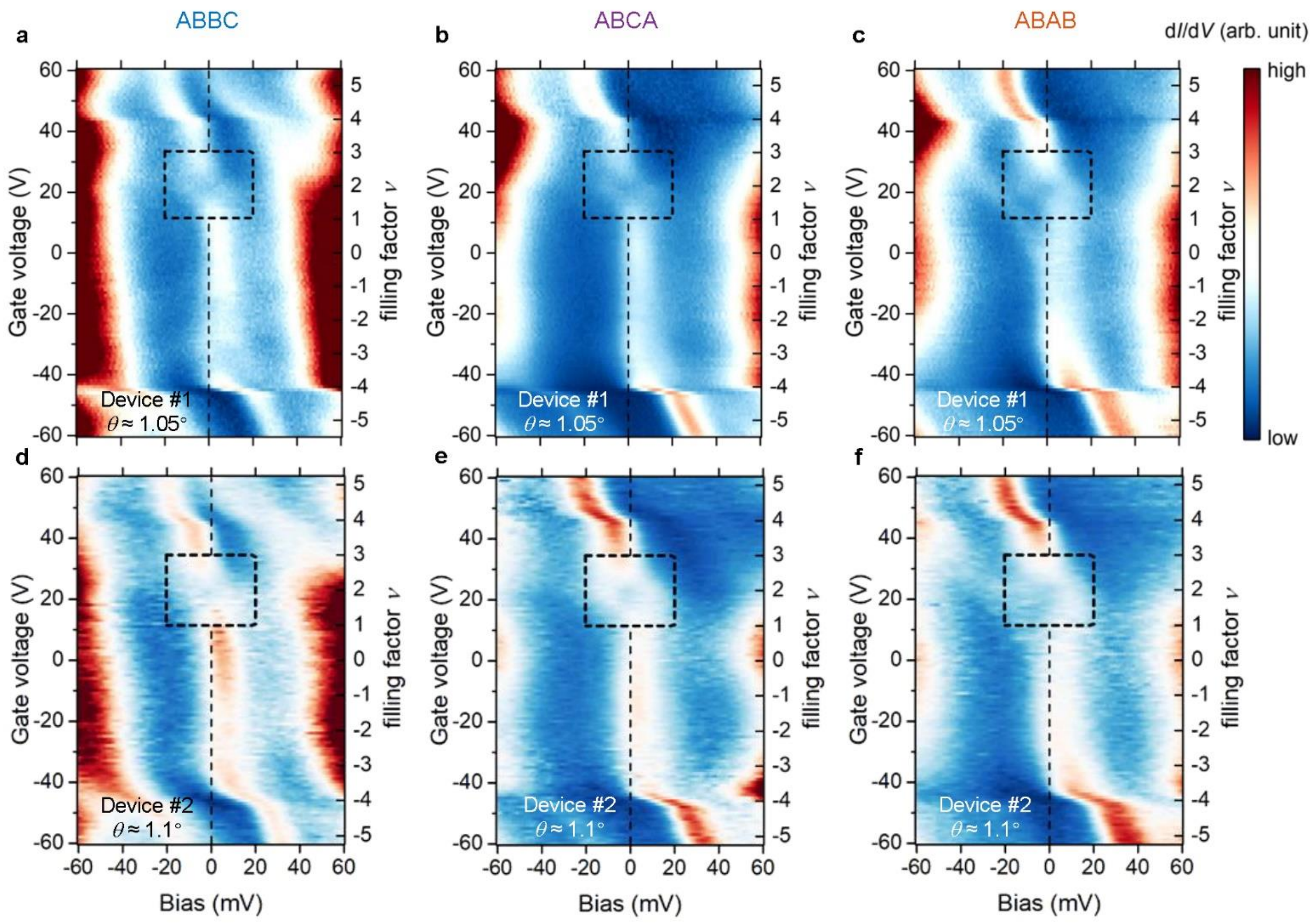

Supplementary Figure 3: Gate-dependent d*I*/d*V* spectroscopy on different devices and/or spots. a-c, Gate-dependent d*I*/d*V* density plot for three stacking regions measured at a different spot on Device #1 (-60 V < V_G < 60 V; modulation voltage V_{RMS} = 0.4 mV; initial V_{Bias} = –100 mV, I_0 = 0.5 nA). The vertical black dashed line denotes zero bias (the Fermi level). The black dashed box highlights the correlation-driven splitting of the FB peak around ν = 2. **d-f**, Same as (**a-c**), but measured on Device #2 (modulation voltage V_{RMS} = 0.4 mV; initial V_{Bias} = 100 mV, I_0 = 0.25 nA).

Supplementary Note 3: Comparison of STM/STS and transport measurements

Transport measurements on tDBLG devices are usually performed with both a top gate and a bottom gate so that the carrier density *n* and the vertical *E*-field can be independently tuned. Results from several groups[2-6] are summarized in the schematic shown in Supplementary Figure 4. A correlated high-resistivity phase surrounded by a "halo" feature can be seen at $\nu = 2$ (labeled as "$n_s/2$"). Signals of incipient insulating states also show up at $\nu = 1, 3$.

In our STM/STS study, on the other hand, only a single back-gate is present (Fig. 1b) and *n* and *E* are always proportionally changed when we adjust V_G (Methods). The parameter space in STM/STS therefore corresponds to a "diagonal" line-cut in the *n*-*E* plane. This line cut touches the half-filling correlated phase but misses many of the other correlation features. Consequently, we only observe splitting of the CFB peak around $\nu = 2$. We note that this limitation of STM/STS can be potentially overcome by using tips with different work functions, thus "moving" the line-cut in the *n*-*E* plane to access other interesting regimes. This is technically extremely challenging, however, and controllable "work function-tunable" tips are currently beyond STM state-of-the-art.

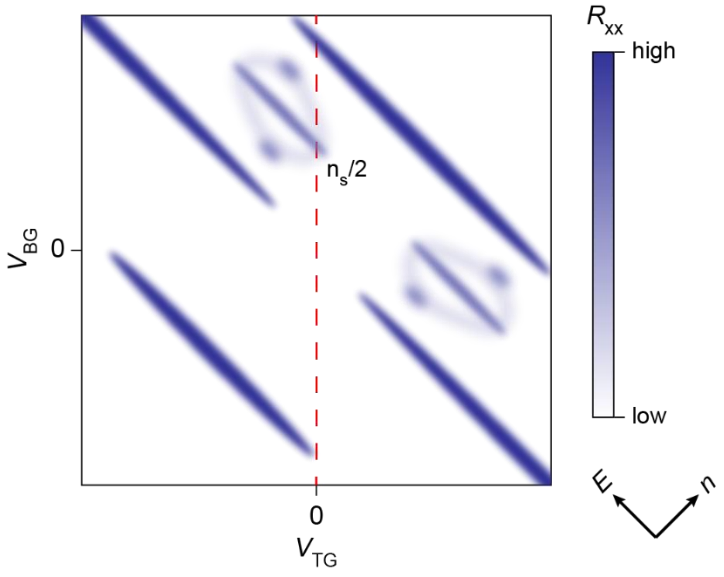

Supplementary Figure 4: Parameter space explored in STM/STS and transport measurements. The red dashed line denotes the parameter space explored in our single-gate STM/STS study.

Supplementary Note 4: Extracting the splitting magnitude from d*I*/d*V* spectra

The magnitude of the energy-splitting was extracted by fitting the d*I*/d*V* spectra with a sum of two Lorentzian peak functions and a linear background

$$y = y_0 + bx + \sum_{i=1}^{2} \frac{2A_i}{\pi} \frac{w}{4\left(x - x_i\right)^2 + w^2} \tag{1}$$

as shown in Supplementary Fig. 5a. Here y_0 and bx are background terms, x_i (i = 1, 2) represents the center position of peak #*i*, A_i is the area under peak #*i*, and w is the peak FWHM. The peak separation $\delta = |x_1 - x_2|$ represents the splitting magnitude. The error bars shown in Fig. 5b were estimated by combining fitting uncertainty, finite temperature broadening, and an instrumental broadening of ~1 mV.

The same fitting procedure was applied to the d*I*/d*V* spectra measured on different devices and/or spots. Supplementary Figure 5b shows the maximum splitting magnitude (squares with error bars) as well as the filling range over which the splitting can be extracted (blue bars) as a function of twist angle θ. More systematic study is required before we can reach a definite conclusion regarding the θ-dependence of the correlation-induced splitting.

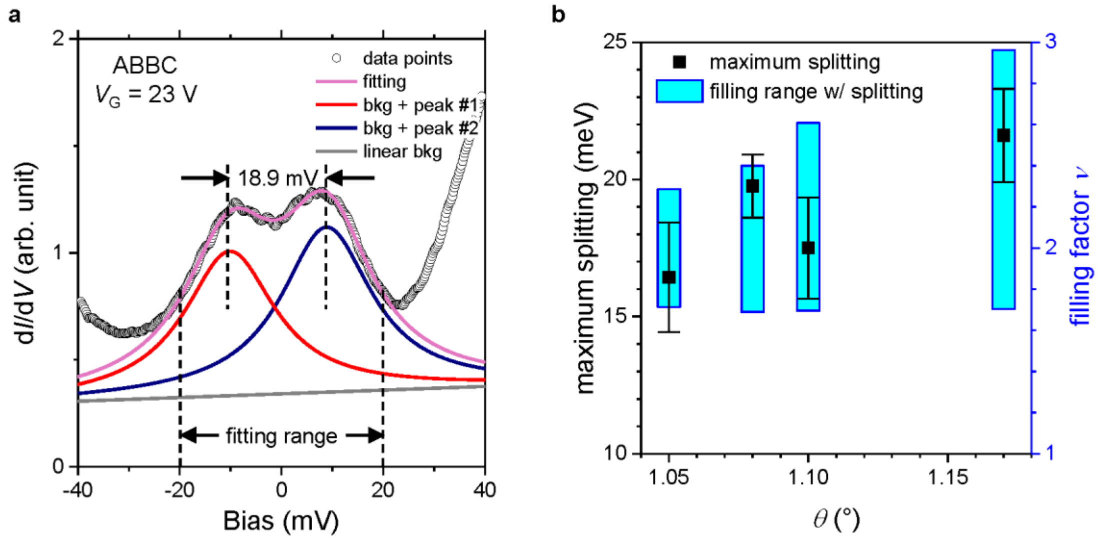

Supplementary Figure 5: Double Lorentzian fitting of the d*I*/d*V* signal. a, Schematic of the fitting procedure. **b**, the maximum splitting magnitude and the filling range in which the splitting can be extracted using this fitting procedure as a function of twist angle θ. The error bars were estimated by combining fitting uncertainty, finite temperature broadening, and an instrumental broadening of ~1 mV.

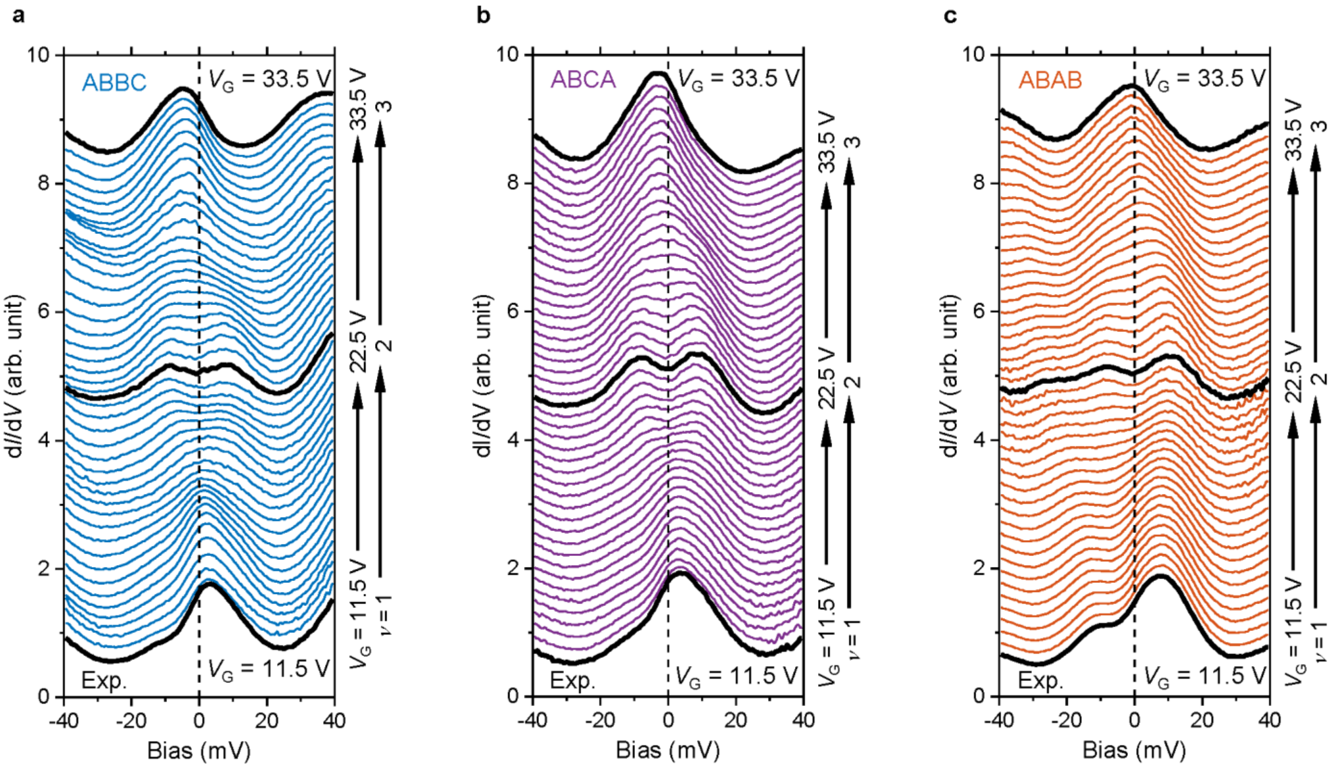

Supplementary Figure 6: dI/dV spectroscopy for three stacking regions for 1 < ν < 3. a-c, Gate-dependent dI/dV spectra for three stacking regions for 11.5 V < V_G < 33.5 V (1 < ν < 3) (modulation voltage V_{RMS} = 1 mV; initial V_{Bias} = –100 mV, I_0 = 0.5 nA).

Supplementary Note 5: Spatial distribution of layer-summed CFB and RCB wavefunctions

The theoretical LDOS shown in Fig. 3 and Supplementary Fig. 2 are projected onto the topmost graphene layer to facilitate direct comparison with STS measurements. To illustrate the localization/delocalization behavior throughout the entire tDBLG stack, we show in Supplementary Fig. 7a the theoretical LDOS at V_G = 60 V and E = 0.33 V/nm for ABBC, ABCA, and ABAB stacking regions when contributions from all four graphene layers are summed. The RCB peak centered at $\mathcal{E}$ = 2.8 meV still only shows up in the ABBC stacking region, and its spatial localization is confirmed by the layer-summed LDOS map (Supplementary Fig. 7c) and the corresponding histogram (Supplementary Fig. 7e) at this energy. The CFB peak centered at $\mathcal{E}$ = –13 meV has significant weight in all three regions, although the intensity in the ABBC region is higher than in the ABCA and ABAB regions. The layer-summed LDOS map at the CFB peak energy shows more spatial variation (Supplementary Fig. 7b,d) compared to the top-layer-projected LDOS map at the same energy (Fig. 3g,i). Nevertheless, the LDOS max/min ratio of 2.5 is still significantly smaller than that of 4.4 in the RCB state, and the CFB remains reasonably delocalized when multiple layers are accounted for.

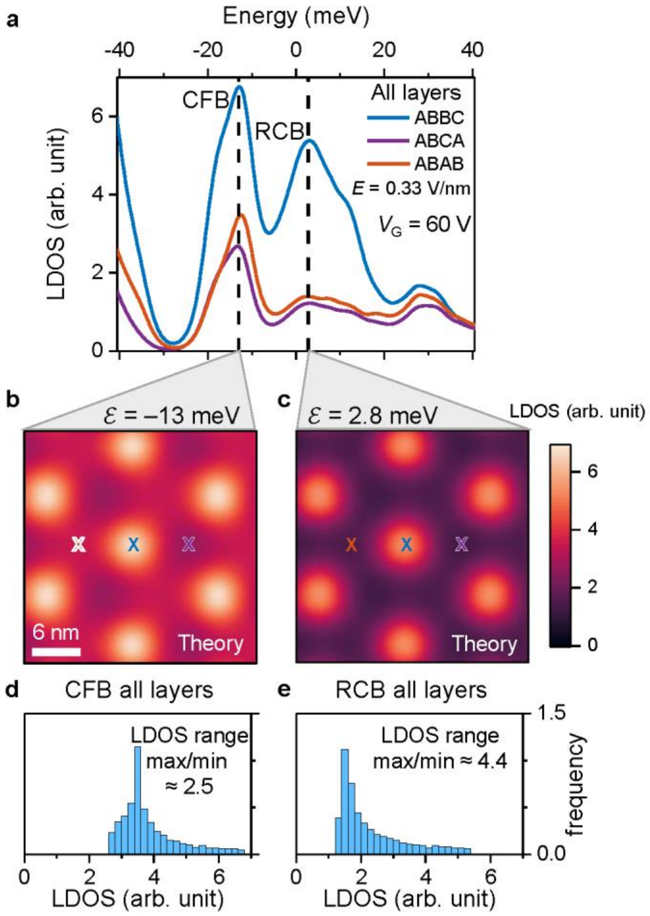

Supplementary Figure 7: Spatial distribution of layer-summed conduction flat band and remote conduction band wavefunctions. a, Theoretical LDOS for three different stacking regions for V_G = 60 V and E = 0.33 V/nm including contributions from all four graphene layers. **b, c**, Layer-summed LDOS maps at (**b**) –13 meV and (**c**) 2.8 meV. **d**, Histogram of (**b**). **e**, Histogram of (**c**). Bin size of 0.2 arb. unit is used. Areas under histograms are normalized to 1. CFB = conduction flat band; RCB = remote conduction band.

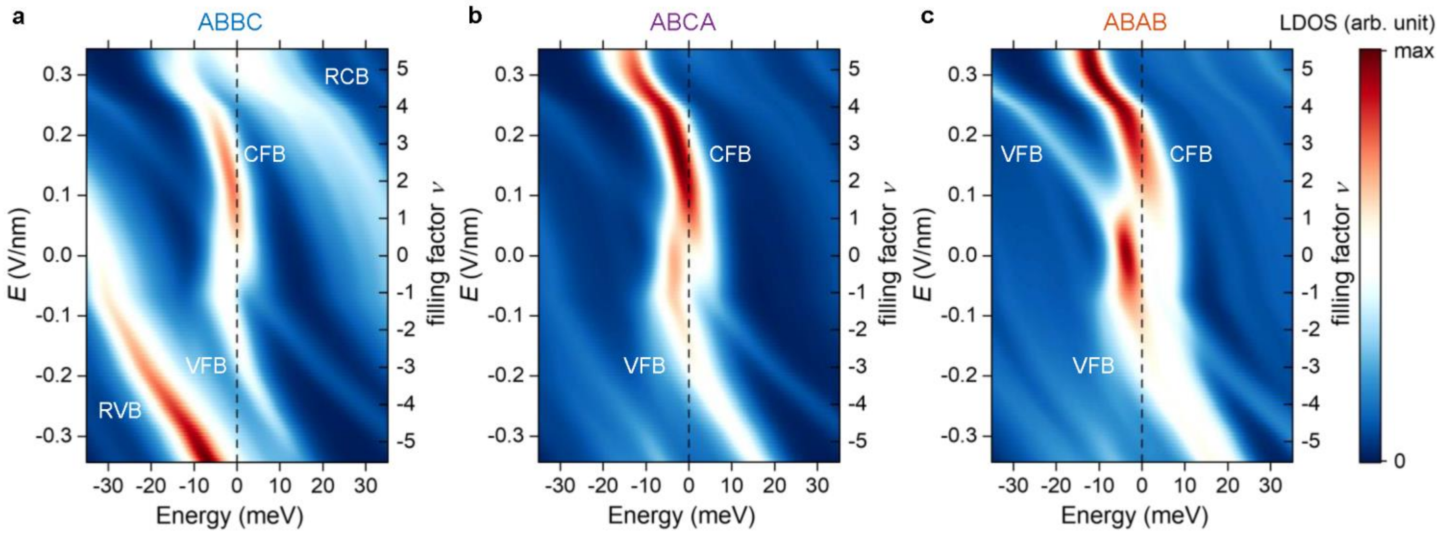

Supplementary Figure 8: Gate-dependent LDOS calculated using a continuum model in the absence of electron-electron interactions. a-c, Density plot of theoretical LDOS (projected onto the top layer) for the three different stacking regions of the moiré unit cell as a function of vertical E-field and filling factor ν. CFB = conduction flat band; VFB = valence flat band; RCB = remote conduction band; RVB = remote valence band.

Supplementary Note 6: Layer polarization of flat band wavefunctions in an *E*-field

The flat band wavefunctions in tDBLG exhibit layer polarization in a gate-induced *E*-field. To illustrate this effect within the single-particle continuum model, we plot the theoretical total DOS as well as LDOS projected onto the topmost graphene layer for both V_G = 27 V, E = +0.15 V/nm (Supplementary Fig. 9a) and V_G = –27 V, E = –0.15 V/nm (Supplementary Fig. 9b). The conduction and valence flat bands appear as two peaks in the total DOS (gray curves). At V_G = 27 V, however, only the CFB peak is prominent in the top-layer LDOS for all three regions. The VFB peak shows up in the ABAB region with much smaller intensity compared to the CFB peak and is absent in the ABBC and ABCA regions. This explains why the VFB signal is experimentally observed only in the ABAB region for positive gate voltage (Fig. 4f). For opposite gate-voltage (V_G = –27 V) the VFB peak shows up clearly in the top layer while the CFB peak is barely present. This explains why experimental d*I*/d*V* spectra measured in the negative gate range do not exhibit the CFB signal for all three stacking regions (Fig. 4d-f).

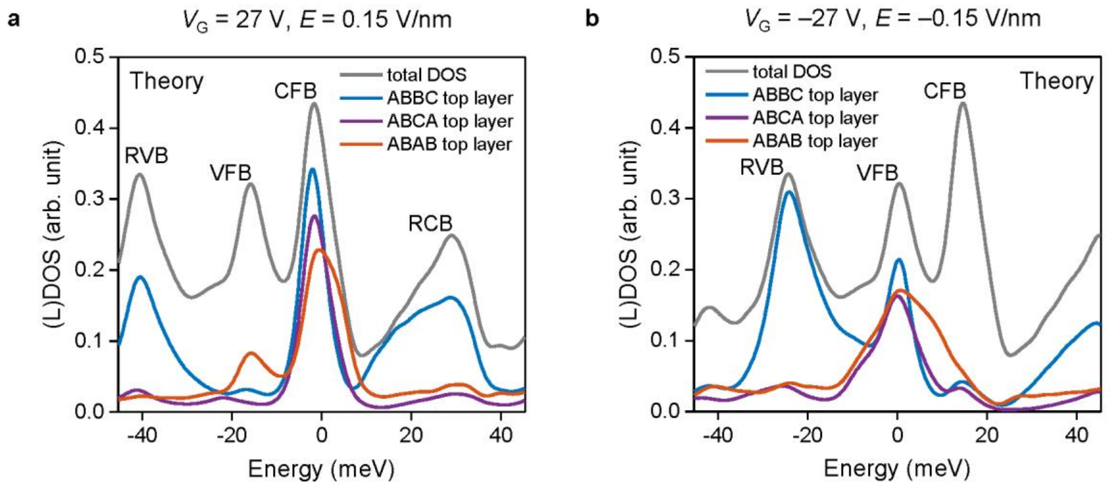

Supplementary Figure 9: Layer polarization of flat band wavefunctions in an *E*-field. a, Theoretical total DOS and LDOS (projected onto the top layer) of three stacking regions at V_G = 27 V and E = 0.15 V/nm. **b**, Same as (**a**), but at V_G = –30 V and E = –0.15 V/nm. CFB = conduction flat band; VFB = valence flat band; RCB = remote conduction band; RVB = remote valence band.

Supplementary Note 7: Screened Coulomb interaction in Hartree-Fock calculations

The single-plane-screened Coulomb potential $V(\boldsymbol{q}) = \frac{e^2}{2\varepsilon_{\text{eff}}\varepsilon_0 q}\left[1-\exp\left(-2qd_{\text{S}}\right)\right]$ in our Hartree-Fock calculations is modeled on the interaction between two bare electrons in the vicinity of a metal plane through a uniform dielectric,[7] a simplification of the measurement setup shown in Fig. 1a. Here we treat the hBN and SiO_2 layers, the other electrons in tDBLG, and the sharp apex of the tungsten STM tip together as the "dielectric", and the macroscopic part of the tip above the apex as the metal plane. The effective screening parameters ε_{eff} and d_{S} can be estimated as follows:

- **Estimation of d_{S}.** Tungsten STM tips prepared by electrochemical etching and field emission typically have an apex of tens of nanometers.[8] In our calculations we take $d_{\text{S}} = 50$ nm, and we note that any value in the range 20 nm $< d_{\text{S}} <$ 100 nm does not cause any qualitative change in the band structure and LDOS.
- **Estimation of ε_{eff}.** Both SiO_2 and hBN have been reported to have dielectric constants between 3 and 7.[9,10] The dielectric constant of tDBLG itself is estimated as $\varepsilon \approx 8$ from Ref.[6]. The tip apex contributes another empirical dielectric factor of 2 to 4 (if we assume a conical shape and solve the Laplace equation to the lowest order). Considering the contributions above, the overall effective dielectric constant lies in the range $3 < \varepsilon_{\text{eff}} < 30$.

Supplementary Note 8: Competition between different symmetry-breaking correlated states

The simplest explanation for reduction in DOS at integer filling is spontaneous

breaking of the spin and valley symmetry. Within Hartree-Fock theory, symmetry breaking is found to be of two distinct types: isospin-polarized (ISP), or inter-valley coherent (IVC).[11] In ISP states the system spontaneously polarizes along an axis in the spin-valley space, producing a multi-component generalization of a ferromagnet (valley polarization induces an orbital magnetic moment). At $\nu = 2$, for example, the possibilities include spin-polarized (e.g. $|K\uparrow\rangle \otimes |K'\uparrow\rangle$), valley-polarized (e.g. $|K\uparrow\rangle \otimes |K\downarrow\rangle$), or spin-valley locked (e.g. $|K\uparrow\rangle \otimes |K'\downarrow\rangle$). Because our model neglects weak intervalley Hund's coupling, there is an enhanced $SU_K(2) \times SU_{K'}(2)$ symmetry for rotating spins independently in each valley, leading to a degeneracy of the various states in the ISP manifold.[11] At integer filling, full polarization can open a charge gap, i.e. a vanishing DOS at the Fermi level (Supplementary Fig. 10a), while away from integer filling the ISP state is analogous to a ferromagnetic metal.

The IVC state, in contrast, spontaneously breaks the valley-U(1) symmetry, and electrons go into a coherent superposition of the two valleys, e.g. $(|K\uparrow,\downarrow\rangle + e^{i\alpha}|K'\uparrow,\downarrow\rangle)/\sqrt{2}$ where α is an angle characterizing the phase of the coherence. At $\nu = 2$, the IVC state does *not* open a gap at the Fermi level; instead, the effective band structure is a semi-metal with a quadratic band touching near the Γ-point (Supplementary Fig. 12a). Nevertheless, due to the large gap away from Γ, the DOS at the Fermi level is reduced compared to the bare single-particle band structure, making it difficult to experimentally distinguish the IVC state from the ISP state through measurement of STS.

In Ref.[11], an analysis of the Hartree-Fock equations at $\nu = 2$ showed that for larger interactions (small ε_{eff}) the S/VP states are preferred, while for weaker interactions (large ε_{eff}) the IVC state is preferred. Here we have numerically solved the Hartree-Fock equations over a continuous range of ν and ε_{eff} to obtain a fuller understanding of the phase diagram. For different ε_{eff} we also proportionally adjust the value of $\varepsilon_{\perp}$(tDBLG) which relates the inter-

layer potential difference U in the continuum model to the physical E-field. To avoid getting stuck in a local minimum, at each ν and ε_{eff} we run the Hartree-Fock using initial ansatzes with distinct symmetry patterns (plus a small amount of noise) and compare the energy of different runs (which sometimes converge to the same state regardless). Here we discuss the results shown in Supplementary Figs. 10-12.

- **Band structure and LDOS at ν = 2.** Supplementary Figures 11a and 12a show the Hartree-Fock band structure at $\nu = 2$ under different screening parameters. For ε_{eff} = 14 the insulating ISP state is favored (Supplementary Fig. 11a; here we plot the spin-polarized band structure as a representative example), while the semi-metallic IVC is the ground state for ε_{eff} = 20 (Supplementary Fig. 12a). In both cases the CFB+ and CFB– peaks as well as the dip feature appear in the LDOS for all three stacking regions (Supplementary Figs. 11b, 12b). The LDOS maps at these peak energies (Supplementary Figs. 11c-d, 12c-d) qualitatively resemble that of the unsplit CFB state (Fig. 3g) and exhibit a small max/min ratio, corroborating our finding of a delocalized correlated state.

- **Phase diagram at ν = 2 and comparison with experiment**. Supplementary Figure 10a shows the energy difference between the ISP and IVC states as a function of ε_{eff}. A crossover from the ISP ground state to the IVC ground state occurs slightly above ε_{eff} = 14. Supplementary Figure 10b shows the effective splitting magnitude of the CFB peak in these two phases. In the main text we utilize ε_{eff} = 14 since this yields the splitting that is closest to the experimental value of ~19 meV.

- **Doping dependence in the range $0 \leq \nu \leq 4$**. Supplementary Figure 11e and 12e show a series of Hartree-Fock DOS for $0 \leq \nu \leq 4$. The CFB peak splits into CFB+ and CFB– for $0.5 \leq \nu \leq 3.25$ at ε_{eff} = 14 (ISP phase) and for $0.75 \leq \nu \leq 2.75$ at ε_{eff} = 20 (IVC

phase). In this doping range the splitting magnitude reaches its maximum at $\nu = 2$ for both the ISP and IVC phases.

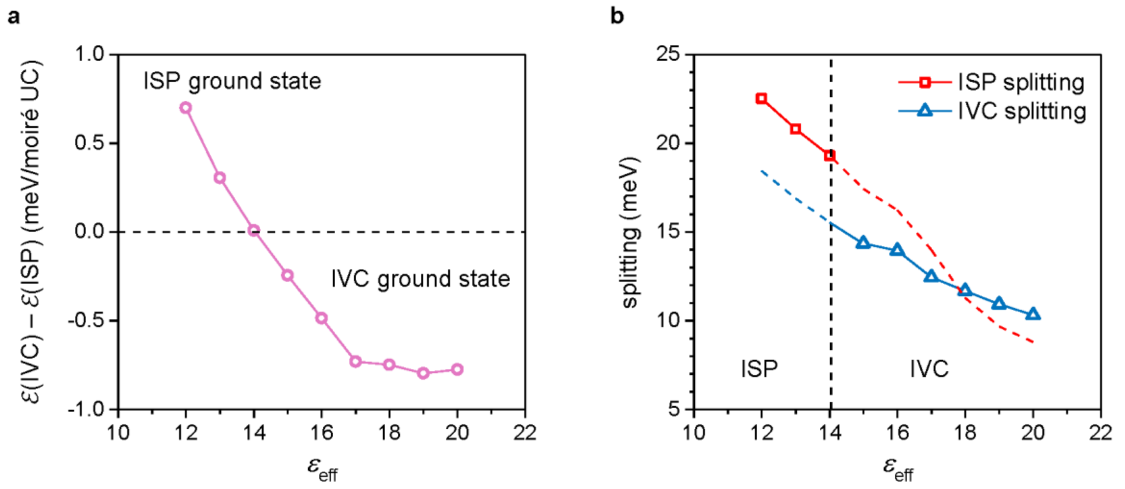

Supplementary Figure 10: Hartree-Fock phase diagram for $\nu = 2$. a, Energy difference between inter-valley coherent (IVC) and isospin-polarized (ISP) states as a function of $\varepsilon_{\mathrm{eff}}$ (d_S = 50 nm). **b**, Magnitude of conduction flat band (CFB) peak-splitting for both ISP and IVC states as a function of $\varepsilon_{\mathrm{eff}}$ (d_S = 50 nm). Dashed lines show energy splitting of the different electronic phases in the dielectric regimes where they are not energetically favorable.

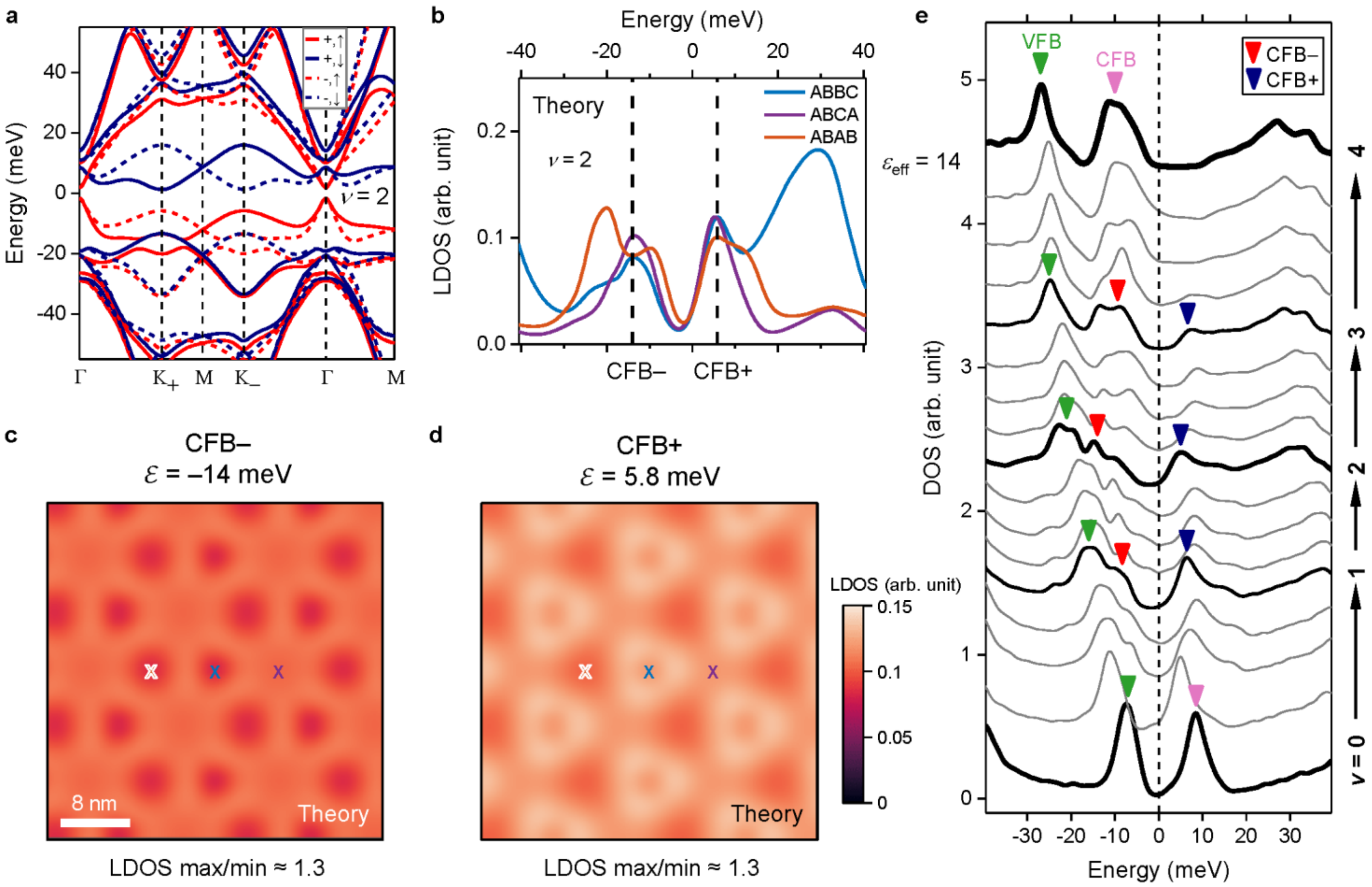

Supplementary Figure 11: Hartree-Fock calculations for d_S = 50 nm and ε_{eff} = 14. a, Hartree-Fock band structure for ν = 2 and E = 0.12 V/nm showing a spin-polarized ground state. "+" and "-" in the legend denote two valleys. **b**, Hartree-Fock LDOS in three different stacking regions for ν = 2 and E = 0.12 V/nm. **c**, **d**, LDOS maps for (**c**) CFB– at –14 meV and (**d**) CFB+ at 5.8 meV. **e**, Hartree-Fock DOS for $0 \leq \nu \leq 4$. CFB = conduction flat band; VFB = valence flat band.

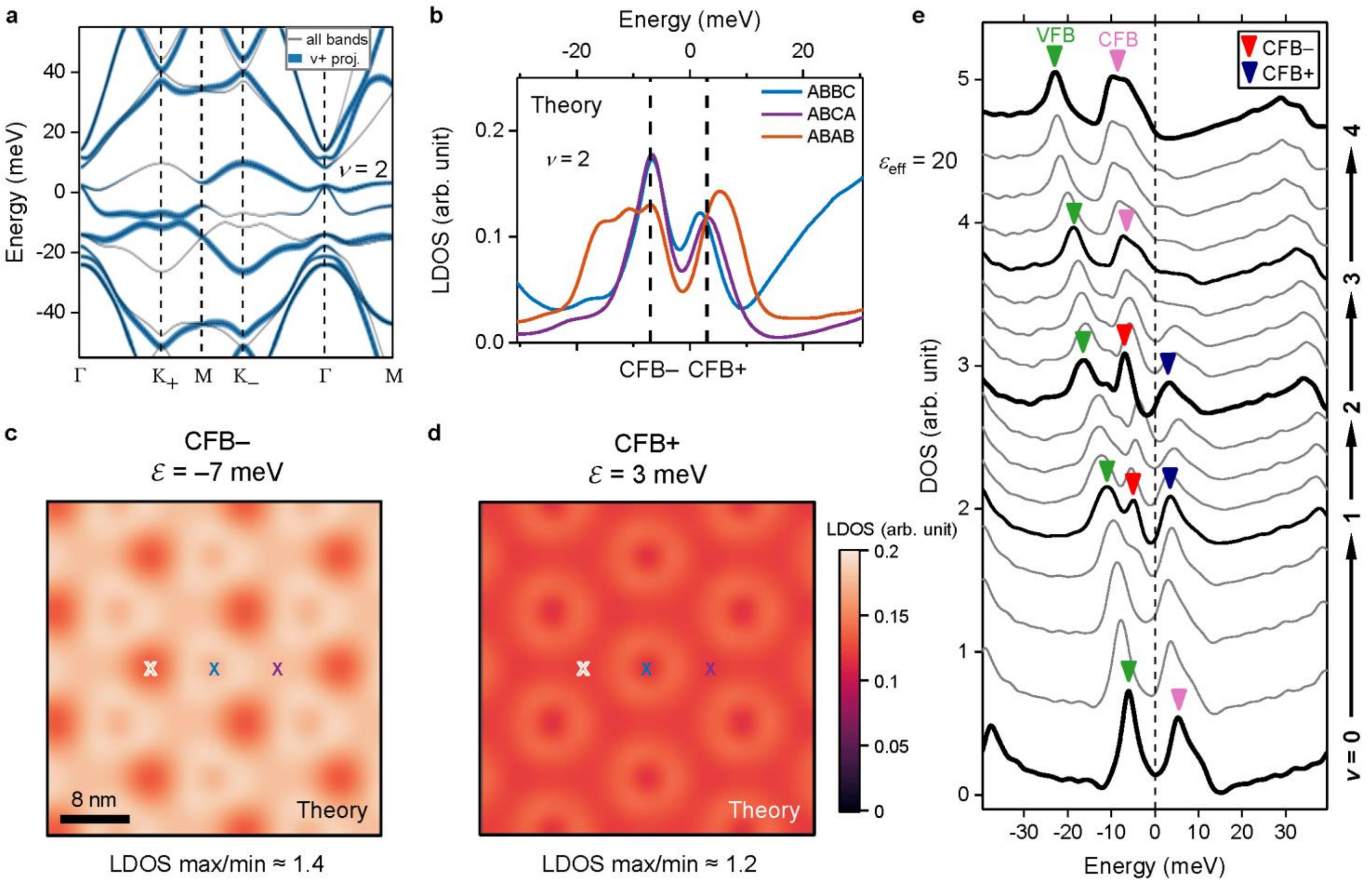

Supplementary Figure 12: Hartree-Fock calculations for d_S = 50 nm and ε_{eff} = 20. a, Hartree-Fock band structure for $\nu = 2$ and E = 0.12 V/nm showing an inter-valley coherent state. The width of the blue lines is proportional to the projection of the wavefunctions onto a certain valley (v+). **b**, Hartree-Fock LDOS in three different stacking regions for $\nu = 2$ and E = 0.12 V/nm. **c**, **d**, LDOS maps for (**c**) CFB–- at –7 meV and (**d**) CFB+ at 3 meV. **e**, Hartree-Fock DOS for $0 < \nu < 4$. CFB = conduction flat band; VFB = valence flat band.

Supplementary Note 9: Tip-bias-induced gating

The presence of a bias voltage across the tip-sample junction can modify both the carrier density n and the E-field in the graphene layers. Here we estimate the tip gating effect by applying the simplest approximation and treating the tip-sample junction as a parallel plate capacitor. We have

$$\Delta n = -\frac{\varepsilon_0 V_{\mathrm{Bias}}}{e d_{\mathrm{T}}} \tag{2}$$

$$\Delta E = \frac{V_{\mathrm{Bias}}}{2 d_{\mathrm{T}}} \tag{3}$$

where V_{Bias} is the sample bias relative to the tip and $d_{\mathrm{T}} \approx 0.7$ nm is the tip-sample distance. Supplementary Figure 13 shows the theoretical LDOS in an ABBC region when these tip-induced corrections are taken into consideration. Since tip-gating is seen to only cause a slight shift in the LDOS curves for the bias range of our measurements, we do not include this effect in the main text. We also note that a more accurate determination of tip-bias-induced corrections would require detailed knowledge of the shape of the tip apex, which is beyond our capability at present.

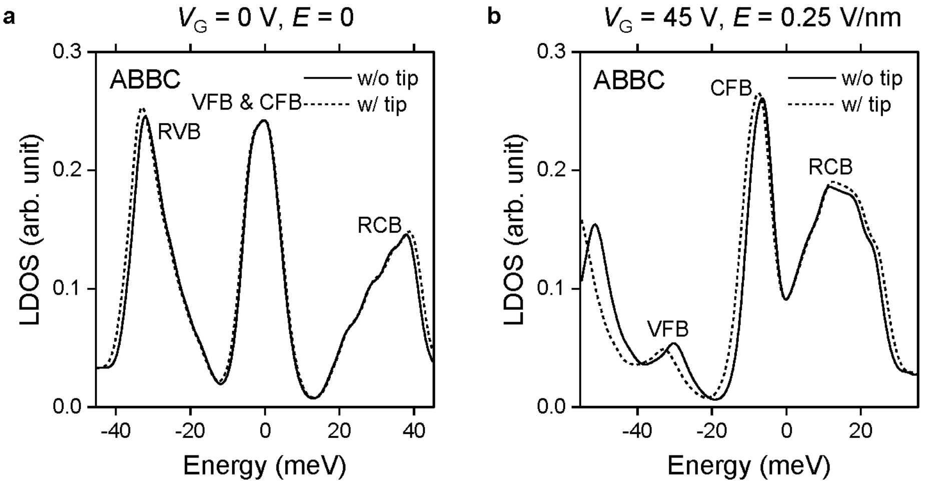

Supplementary Figure 13: Theoretical LDOS including tip-bias-induced gating. a, Theoretical LDOS in an ABAB region at V_G = 0 V and E = 0 with and without correction terms due to tip-bias-induced gating. **b**, same as (**a**), but for V_G = 45 V and E = 0.25 V/nm. CFB = conduction flat band; VFB = valence flat band; RCB = remote conduction band; RVB = remote valence band.

References

1 Choi, Y. W. & Choi, H. J. Intrinsic band gap and electrically tunable flat bands in twisted double bilayer graphene. *Physical Review B* **100**, 201402, doi:10.1103/PhysRevB.100.201402 (2019).

2 Burg, G. W. *et al.* Correlated Insulating States in Twisted Double Bilayer Graphene. *Physical Review Letters* **123**, 197702, doi:10.1103/PhysRevLett.123.197702 (2019).

3 Shen, C. *et al.* Correlated states in twisted double bilayer graphene. *Nature Physics* **16**, 520-525, doi:10.1038/s41567-020-0825-9 (2020).

4 Liu, X. *et al.* Tunable spin-polarized correlated states in twisted double bilayer graphene. *Nature* **583**, 221-225, doi:10.1038/s41586-020-2458-7 (2020).

5 Cao, Y. *et al.* Tunable correlated states and spin-polarized phases in twisted bilayer–bilayer graphene. *Nature* **583**, 215-220, doi:10.1038/s41586-020-2260-6 (2020).

6 He, M. *et al.* Symmetry breaking in twisted double bilayer graphene. *Nature Physics* **17**, 26-30, doi:10.1038/s41567-020-1030-6 (2021).

7 Bultinck, N. *et al.* Ground State and Hidden Symmetry of Magic-Angle Graphene at Even Integer Filling. *Physical Review X* **10**, 031034, doi:10.1103/PhysRevX.10.031034 (2020).

8 Ernst, S., Wirth, S., Rams, M., Dolocan, V. & Steglich, F. Tip preparation for usage in an ultra-low temperature UHV scanning tunneling microscope. *Science and Technology of Advanced Materials* **8**, 347-351, doi:10.1016/j.stam.2007.05.008 (2007).

9 Gray, P. R., Hurst, P., Meyer, R. G. & Lewis, S. *Analysis and design of analog integrated circuits*. (Wiley, 2001).

10 Laturia, A., Van de Put, M. L. & Vandenberghe, W. G. Dielectric properties of hexagonal boron nitride and transition metal dichalcogenides: from monolayer to bulk. *npj 2D Materials and Applications* **2**, 6, doi:10.1038/s41699-018-0050-x (2018).

11 Lee, J. Y. *et al.* Theory of correlated insulating behaviour and spin-triplet superconductivity in twisted double bilayer graphene. *Nature Communications* **10**, 5333, doi:10.1038/s41467-019-12981-1 (2019).
